# Single-center experience on catheter-related sepsis in tunneled central venous catheters in hemodialysis patients 

**DOI:** 10.5414/CNP104S08

**Published:** 2025-11-28

**Authors:** Tjaša Furlan, Karmen Terbovc, Boštjan Leskovar

**Affiliations:** Vascular Disease and Vascular Access Unit, Trbovlje General Hospital, Trbovlje, Slovenia

**Keywords:** tunneled central venous catheter, catheter-related bloodstream infection, catheter-related sepsis, tunnel infections, hemodialysis

## Abstract

Introduction: Catheter-related infections significantly increase the morbidity and mortality of dialysis patients. Our study aimed to assess the incidence of catheter-related infections in tunneled central venous catheters at our dialysis center. Materials and methods: We retrospectively analyzed the incidence of catheter-related bloodstream infections (CRBSI) and tunnel infections in patients with a tunneled central venous catheter inserted at Trbovlje General Hospital between January 2015 and August 2024. The indication for catheter insertion was a reduced left ventricular ejection fraction (< 30%), polymorbidity with a short life expectancy (< 1 year), or a condition of the vascular system that made construction of an arteriovenous fistula/graft impossible. Results: We included 344 patients (average age 73 ± 13 years, 49% men). In all patients, we inserted a tunneled central venous catheter with a symmetrical tip in a retrograde manner (64% through the right jugular vein). During the observed period, there were 16 cases of CRBSI (after 10 (IQR 5 – 14) months) and 4 cases of catheter tunnel infection (after 9 (IQR 4 – 16) months). In all cases of CRBSI, the catheter was replaced. In the case of catheter tunnel infections, the outer part of the catheter was replaced, and the tunnel infection was treated locally. The incidence of CRBSI was 0.09/1,000 catheter days. Conclusion: The incidence of CRBSI in our cohort was low, likely due to the implementation of preventive protocols for insertion and maintenance of tunneled central venous catheters.

## Introduction 

A tunneled central venous catheter can be used for short- and intermediate-term vascular access in patients without a functional arteriovenous fistula/graft or as permanent vascular access in patients with limited alternative options [1, 2]. The National Kidney Foundation Kidney Disease Outcomes Quality Initiative (NKF-KDOQI) guidelines recommend using a tunneled catheter if a “temporary” catheter is needed for more than 3 weeks [2]. 

One of the most critical complications of central venous catheters is catheter-related infection, which significantly increases the morbidity and mortality of dialysis patients [3]. Catheter-related infections in tunneled central venous catheters can be divided into catheter-related sepsis (catheter-related bloodstream infection (CRBSI)) and tunnel infections. 

Many interventions have been introduced to reduce the risk of CRBSI, including antimicrobial lock solutions [4] and collaborative preventive measures [5, 6]. Clear nursing instructions during discharge are crucial for patients and the medical staff. Simultaneously, the patency and occurrence of catheter-related infections in patients with inserted catheters must be monitored. 

This study presents our results on catheter-related infections in tunneled central venous catheters in a period of approximately 9 years. 

## Materials and methods 

In this retrospective study, we analyzed the incidence, causative agents, and treatment of catheter-related infections. We included patients with a tunneled central venous catheter inserted at the Vascular Disease and Vascular Access Unit, Trbovlje General Hospital, Trbovlje, Slovenia, between January 2015 and August 2024. The indication for a tunneled central venous catheter insertion in patients with end-stage kidney disease was a reduced left ventricular ejection fraction (< 30%), polymorbidity with a short life expectancy (< 1 year), or a condition of the vascular system where arteriovenous fistula or graft construction was not possible. 

All catheters were inserted in the operating room in aseptic conditions by a nephrologist experienced in catheter insertion and percutaneous endovascular procedures. The vein was punctured under ultrasound guidance, and the catheter was inserted and positioned under fluoroscopy guidance. The position of the catheter was confirmed by X-ray before the next hemodialysis, which was done in our center before discharge. We used retrograde tunneled central venous catheters with a symmetrical tip (Arrow-Clark, VectorFlow hemodialysis catheter, Teleflex, Morrisville, NC, USA). 

Patients and medical personnel received written instructions on caring for their tunneled central venous catheter at discharge. We advised the following protocol that we also use in our dialysis center:

- Regular alternating use of bactericidal and thrombolytic filling solutions (use of 30% citrate for regular filling of the catheter and use of alteplase every 3^rd^ – 6^th^ filling of the catheter);
- Use of antibacterial caps;
- Covering the exit site of the catheter with air-permeable dressings or not covering the catheter at all after shaping of the catheter exit site;
- Strict aseptic care of the catheter, including using a face mask and sterile gloves at all times when handling the opened catheter Luer-Lock connectors.

Data on the patient’s tunneled catheter conditions were collected from our local and central national medical databases, as well as the host hemodialysis center for patients with missing or unavailable data. The Institute of Microbiology and Immunology, Faculty of Medicine, University of Ljubljana, Slovenia, and the National Institute for Public Health, Ljubljana, Slovenia, provided the data on causative agents. 

We analyzed the incidence, treatment, and outcomes of CRBSI and tunnel infections during the study period. Tunnel infections and CRBSI were defined according to the NKF-KDOQI Clinical Practice Guideline for Vascular Access [2]:

- CRBSI: clinical manifestations and at least 1 positive blood culture from a peripheral source and no other apparent source, with either positive semiquantitative (> 15 colony-forming unit/catheter segment, hub or tip) or quantitative (> 102 colony-forming unit/catheter segment, hub or tip) culture, whereby the same organism (species and antibiogram) is isolated from the catheter segment (hub or tip) and a peripheral source blood sample;
- Tunnel infection: tenderness, hyperemia, and/or induration that extends along the subcutaneous tunnel. It may or may not be associated with bacteremia.

Statistical analyses were performed with STATA, version 17.0 (StataCorp LLC, College Station, TX, USA). Continuous variables are reported as mean ± standard deviation or median and interquartile range (IQR), while categorical variables are reported as frequencies (percentages). Frequency of CRBSI and tunnel infections is reported as an incidence rate per 1,000 catheter days. 

## Results 

We included 344 patients, 49% were male, with an average age of 73 ± 13 years. In all patients, we inserted a tunneled central venous catheter in a retrograde manner, in 215 (62.5%) cases through the right jugular vein, in 123 (35.8%) cases through the left jugular vein, and in 6 (1.7%) instances through the right subclavian vein. The median observation period was 11 (IQR 4 – 108) months. 

During the study period, we observed 16 cases of CRBSI (after a median of 10 (IQR 5 – 14) months) and 4 cases of catheter tunnel infection (after a median of 9 (IQR 4 – 16) months). The most common causative agent of infection was methicillin-susceptible *Staphylococcus aureus* in CRBSI and methicillin-susceptible *Staphylococcus epidermidis* in tunnel infection (Table 1). 

The incidence of CRBSI was 0.09/1,000 catheter days, and the incidence of tunnel infection was 0.02/1,000 catheter days. We recorded 4 cases of infectious endocarditis with CRBSI; other metastatic infectious complications, such as osteomyelitis, septic arthritis, or epidural abscess, were not observed. Mortality in patients with CRBSI was 4/16 (25%). All patients who died because of CRBSI had concomitant infectious endocarditis. 

The treatment of CRBSI and tunnel infections was according to the current NKF-KDOQI guidelines [2]. In the case of CRBSI, the catheter was replaced over the guidewire with a new tunnel formation (immediately or postponed) or removed and reinserted on the opposite side, depending on the isolated pathogen and the patient’s condition. In the case of tunnel infection, the outer part of the retrograde catheter was replaced, and the tunnel infection was treated both locally, through exit site incision and local disinfection, and systemically, with antibiotics. Despite some recommendations that the dialysis catheter should be removed in these cases [7], we treated all our tunnel infections with replacement of the outer part of the catheter and local tunnel treatment with regular injections of disinfectants and local antibiotics (preferably gentamicin) into the tunnel (provided that the repeat blood cultures were negative). In cases of concomitant cellulitis without cuff involvement, we incised the distal part of the tunnel and treated the patient with systemic antibiotics. The tunnel infection was successfully cured in all cases, and we observed no progression to systemic infection. 

## Discussion 

Our single-center retrospective analysis of 344 hemodialysis patients with tunneled central venous catheters revealed a low incidence of CRBSI and tunnel infections over approximately 9 years. 

One of the most critical complications of central venous catheters is catheter-related infection, which significantly increases the morbidity and mortality of dialysis patients [2]. Most infections are due to skin flora colonizing the site of catheter placement [8]. The incidence of hemodialysis catheter-related sepsis reported in the literature is ~ 0.35 – 0.73/1,000 catheter days [9, 10, 11]. Our reported incidence of CRBSI and tunnel infections is much lower than previously described. Metastatic infections, such as osteomyelitis, endocarditis, septic arthritis, or epidural abscess have been observed in ~ 5 – 10% of catheter-dependent hemodialysis patients [12], most frequently in association with *Staphylococcus aureus* infections [14, 15]. We observed 4 cases of infectious endocarditis in our patients (*Staphylococcus aureus* was the causative agent in 2 cases, methicillin-resistant *Staphylococcus aureus* and methicillin-resistant *Staphylococcus epidermidis* each in 1 case). Metastatic infections can be minimized by prompt removal of the catheter [8, 16], but cannot always be prevented, especially in infections with invasive and antibiotic-resistant bacteria. 

The most common causative pathogens of catheter-related infections are Gram-positive organisms, with *Staphylococcus aureus* and coagulase-negative *Staphylococci* accounting for 40 – 80% of cases [10, 14, 17, 18, 19, 20]. Gram-negative organisms account for 20 – 40%, and polymicrobial infections for 10 – 20% of all episodes of catheter-related infections [9]. In accordance with prior studies, our study identified methicillin-susceptible *Staphylococcus aureus* as the most common causative agent in catheter-related sepsis. In contrast, methicillin-susceptible *Staphylococcus epidermidis*, the most common organism of skin flora, was the most common causative agent of tunnel infection. 

The management of catheter-related sepsis includes antimicrobial therapy and catheter management [2, 15]. Empiric systemic antimicrobial therapy should be initiated immediately after obtaining blood cultures in patients suspected of having catheter-related sepsis. If immediate catheter removal is not possible, it should be treated with antibiotic lock therapy. All our patients with catheter-related sepsis were treated with systemic antibiotics and catheter removal. 

Prevention of catheter-related infections is at least as important as the treatment [2, 15]. General preventive measures to reduce catheter-related infections include written protocols with detailed instructions on the use of aseptic techniques when handling the catheter, accompanied by strict aseptic techniques and the use of chlorhexidine or alcohol-coated catheter caps. Several studies have confirmed a reduced rate of catheter-related infections when using alcohol or chlorhexidine-coated catheter caps, with no reported device-related adverse events [20]. Using taurolidine- or alteplase-containing catheter lock solutions has shown antimicrobial properties [21, 22, 23, 24, 25]. We propose that the rate of catheter-related infections at our vascular access center is low precisely because of the strict preventive measures that were in place. All catheters were inserted in the operating room by ultrasound and X-ray guidance, and patients also received written instructions on caring for their tunneled central venous catheter at discharge to their dialysis center. 

The main limitation of our study is that it is a retrospective, single-center study. Additional limitations include the lack of comparison between tunneled catheters and temporary catheters for permanent use, no quality-of-life analysis, and no flow patency analysis. 

To conclude, strict aseptic measures during tunneled catheter insertion, nursing care in the first period until the exit site is formed, and strict aseptic measures while Luer-Lock connections are open are essential in preventing catheter-related infections. Combining the nursing protocol of locking solutions and antimicrobial caps with a follow-up program for patients with permanent central catheter dialysis as permanent vascular access are key aspects that prevent infection. 

## Compliance with ethical standards 

The Medical Ethics Committee approved the study (EK08/2024). Due to its retrospective nature, the patient’s consent was waived. 

## Acknowledgments 

The authors thank the Hemodialysis and Internal Medicine Department at Trbovlje General Hospital for their support and cooperation in the vascular access program. 

## Authors’ contributions 

T.F. and B.L. conceived and designed the study. T.F. and K.T. collected the data and T.F. performed the statistical analysis. T.F. wrote the manuscript. B.L. read and critically evaluated the manuscript and gave final approval for publication. 

## Funding 

The authors received no financial support for this article’s research, authorship, and/or publication. 

## Conflict of interest 

The authors declare no potential conflict of interest concerning this article’s research, authorship, and/or publication. 

**Table 1.** Causative agents of catheter-related infections.

Catheter-related bloodstream infection		Tunnel infection	
Causative infectious agent	N of cases	Causative infectious agent	N of cases
MSSA	3	MSSE	2
MSSE	2	Unidentified	2
*Stenotrophomonas maltophilia*	2		
MRSA	2		
MSSA and *Corynebacterium striatum*	2		
MRSE	1		
*Escherichia coli* and *Enterococcus faecalis*	1		
*Enterococcus faecalis* and *Bacteroides fragilis*	1		
*Enterobacter cloacae* complex	1		
*Pseudomonas aeruginosa*	1		

N = number; MSSA = methicillin-susceptible *Staphylococcus aureus*; MSSE = methicillin-susceptible *Staphylococcus epidermidis*; MRSA = methicillin-resistant *Staphylococcus aureus*; MRSE = methicillin-resistant *Staphylococcus epidermidis*.

## References

[1] Hemodialysis Adequacy 2006 Work Group. Clinical practice guidelines for hemodialysis adequacy, update 2006. Am J Kidney Dis. 2006; 48: S2–S90. 16813990 10.1053/j.ajkd.2006.03.051

[2] Lok CE Huber TS Lee T Shenoy S Yevzlin AS Abreo K Allon M Asif A Astor BC Glickman MH Graham J Moist LM Rajan DK Roberts C Vachharajani TJ Valentini RP 2019 Update. Am J Kidney Dis. 2020; 75: S1–S164. 32778223 10.1053/j.ajkd.2019.12.001

[3] Kumbar L Yee J Current Concepts in Hemodialysis Vascular Access Infections. Adv Chronic Kidney Dis. 2019; 26: 16–22. 30876612 10.1053/j.ackd.2018.10.005

[4] Arechabala MC Catoni MI Claro JC Rojas NP Rubio ME Calvo MA Letelier LM Antimicrobial lock solutions for preventing catheter-related infections in haemodialysis. Cochrane Database Syst Rev. 2018; 4: CD010597. 29611180 10.1002/14651858.CD010597.pub2 PMC6513408

[5] Patel PR Yi SH Booth S Bren V Downham G Hess S Kelley K Lincoln M Morrissette K Lindberg C Jernigan JA Kallen AJ Yi SH Booth S Bren V Downham G Hess S Kelley K Lincoln M Morrissette K Lindberg C Jernigan JA Kallen AJ. Bloodstream infection rates in outpatient hemodialysis facilities participating in a collaborative prevention effort: a quality improvement report. Am J Kidney Dis. 2013; 62: 322–330. 23676763 10.1053/j.ajkd.2013.03.011

[6] Rosenblum A Wang W Ball LK Latham C Maddux FW Lacson E Hemodialysis catheter care strategies: a cluster-randomized quality improvement initiative. Am J Kidney Dis. 2014; 63: 259–267. 24295613 10.1053/j.ajkd.2013.08.019

[7] Beathard GA Management of bacteremia associated with tunneled-cuffed hemodialysis catheters. J Am Soc Nephrol. 1999; 10: 1045–1049. 10232691 10.1681/ASN.V1051045

[8] El Khudari H Ozen M Kowalczyk B Bassuner J Almehmi A Hemodialysis Catheters: Update on Types, Outcomes, Designs and Complications. Semin Intervent Radiol. 2022; 39: 90–102. 35210738 10.1055/s-0042-1742346 PMC8856777

[9] Allon M Dialysis catheter-related bacteremia: treatment and prophylaxis. Am J Kidney Dis. 2004; 44: 779–791. 15492943

[10] Almenara-Tejederas M Rodríguez-Pérez MA Moyano-Franco MJ de Cueto-López M Rodríguez-Baño J Salgueira-Lazo M Tunneled catheter-related bacteremia in hemodialysis patients: incidence, risk factors and outcomes. A 14-year observational study. J Nephrol. 2023; 36: 203–212. 35976569 10.1007/s40620-022-01408-8 PMC9895018

[11] Mandolfo S Possenti S Lucca B Bracchi M Bove S Bertelli C Costantino E Alberici F Tunneled hemodialysis central venous catheters prevalence and bloodstream infection rates in Northern Italy: A survey of the “East Lombardy Nephrological Network”. J Vasc Access. 2024; 25: 2001–2006. 37861341 10.1177/11297298231202081

[12] Farrington CA Allon M Complications of Hemodialysis Catheter Bloodstream Infections: Impact of Infecting Organism. Am J Nephrol. 2019; 50: 126–132. 31242483 10.1159/000501357 PMC6935870

[13] Marr KA Sexton DJ Conlon PJ Corey GR Schwab SJ Kirkland KB Catheter-related bacteremia and outcome of attempted catheter salvage in patients undergoing hemodialysis. Ann Intern Med. 1997; 127: 275–280. 9265426 10.7326/0003-4819-127-4-199708150-00003

[14] Maya ID Carlton D Estrada E Allon M Treatment of dialysis catheter-related Staphylococcus aureus bacteremia with an antibiotic lock: a quality improvement report. Am J Kidney Dis. 2007; 50: 289–295. 17660030 10.1053/j.ajkd.2007.04.014

[15] Mermel LA Allon M Bouza E Craven DE Flynn P O’Grady NP Raad II Rijnders BJ Sherertz RJ Warren DK Clinical practice guidelines for the diagnosis and management of intravascular catheter-related infection: 2009 Update by the Infectious Diseases Society of America. Clin Infect Dis. 2009; 49: 1–45. 19489710 10.1086/599376 PMC4039170

[16] Cheesbrough JS Finch RG Burden RP A prospective study of the mechanisms of infection associated with hemodialysis catheters. J Infect Dis. 1986; 154: 579–589. 3745972 10.1093/infdis/154.4.579

[17] Almirall J Gonzalez J Rello J Campistol JM Montoliu J Puig de la Bellacasa J Revert L Gatell JM Infection of hemodialysis catheters: incidence and mechanisms. Am J Nephrol. 1989; 9: 454–459. 2596535 10.1159/000168012

[18] Swartz RD Messana JM Boyer CJ Lunde NM Weitzel WF Hartman TL Successful use of cuffed central venous hemodialysis catheters inserted percutaneously. J Am Soc Nephrol. 1994; 4: 1719–1725. 8011982 10.1681/ASN.V491719

[19] Jacobsson G Dashti S Wahlberg T Andersson R The epidemiology of and risk factors for invasive Staphylococcus aureus infections in western Sweden. Scand J Infect Dis. 2007; 39: 6–13. 17366006 10.1080/00365540600810026

[20] Brunelli SM Van Wyck DB Njord L Ziebol RJ Lynch LE Killion DP Cluster-Randomized Trial of Devices to Prevent Catheter-Related Bloodstream Infection. J Am Soc Nephrol. 2018; 29: 1336–1343. 29472415 10.1681/ASN.2017080870 PMC5875956

[21] Olthof ED Rentenaar RJ Rijs AJMM Wanten GJA Absence of microbial adaptation to taurolidine in patients on home parenteral nutrition who develop catheter related bloodstream infections and use taurolidine locks. Clin Nutr. 2013; 32: 538–542. 23267744 10.1016/j.clnu.2012.11.014

[22] Weck S Cheung S Hiraoka-Sutow M Patapoff T Semba CP Alteplase as a catheter locking solution: in vitro evaluation of biochemical stability and antimicrobial properties. J Vasc Interv Radiol. 2005; 16: 379–383. 15758134 10.1097/01.RVI.0000148154.30967.27

[23] Onder AM Chandar J Simon N Saint-Vil M Francoeur D Nwobi O Abitbol C Zilleruelo G Treatment of catheter-related bacteremia with tissue plasminogen activator antibiotic locks. Pediatr Nephrol. 2008; 23: 457–464. 18064496 10.1007/s00467-007-0687-8

[24] Onder AM Chandar J Billings A Simon N Gonzalez J Francoeur D Abitbol C Zilleruelo G Prophylaxis of catheter-related bacteremia using tissue plasminogen activator-tobramycin locks. Pediatr Nephrol. 2009; 24: 2233–2243. 19590902 10.1007/s00467-009-1235-5

[25] Hemmelgarn BR Moist LM Lok CE Tonelli M Manns BJ Holden RM LeBlanc M Faris P Barre P Zhang J Scott-Douglas N Prevention of dialysis catheter malfunction with recombinant tissue plasminogen activator. N Engl J Med. 2011; 364: 303–312. 21268722 10.1056/NEJMoa1011376

